# Similarity analysis of federal reserve statements using document embeddings: the Great Recession vs. COVID-19

**DOI:** 10.1007/s43546-022-00248-9

**Published:** 2022-06-18

**Authors:** Luis Felipe Gutiérrez, Neda Tavakoli, Sima Siami-Namini, Akbar Siami Namin

**Affiliations:** 1 Texas Tech University, Lubbock, TX USA; 2 Georgia Institute of Technology, Atlanta, GA USA; 3 Rutgers University, New Brunswick, NJ USA

## Abstract

The coronavirus pandemic has already caused plenty of severe problems for humanity and the economy. The exact impact of the COVID-19 pandemic is still unknown, and economists and financial advisers are exploring all possible scenarios to mitigate the risks arising from the pandemic. An intriguing question is whether this pandemic and its impacts are similar, and to what extent, to any other catastrophic events that occurred in the past, such as the 2009 Great Recession. This paper intends to address this problem by analyzing official public announcements and statements issued by federal authorities such as the Federal Reserve. More specifically, we measure similarities of consecutive statements issued by the Federal Reserve during the 2009 Great Recession and the COVID-19 pandemic using natural language processing techniques. Furthermore, we explore the usage of document embedding representations of the statements in a more complex task: clustering. Our analysis shows that, using an advanced NLP technique in document embedding such as Doc2Vec, we can detect a difference of 10.8% in similarities of Federal Open Market Committee (FOMC) statements issued during the Great Recession (2007–2009) and the COVID-19 pandemic. Finally, the results of our clustering exercise show that the document embeddings representations of the statements are suitable for more complex tasks, which provides a basis for future applications of state-of-the-art natural language processing techniques using the FOMC post-meeting statements as the dataset.

## Introduction

The 2009 Great Recession struck the world financial markets and banking systems very severely. It caused millions of people to lose their jobs and homes.

The consequences of this financial crisis were so severe in the United States more than anywhere else on the planet due to banking problems in mortgages and loans. It took six years for the US economy to recover from the Great Recession to where it was at the beginning of the recession (Chart book: The legacy of the great recession 2019).

After a decade, the world witnessed the widespread of a deadly and novel virus that caused a contagious disease called COVID-19. Today, the virus has taken over 700,000 lives worldwide and about 160,000 from the U.S only. In addition to being a threat to humanity, the economic impact of this unwanted virus is still unknown. According to a recent study (Han et al. 2020), this pandemic has negatively affected income and thus increased poverty.

The Great Recession of 2009 and COVID-19 have some similarities, but also they exhibit a different level of uncertainty for government and economic policy-makers. In the 2009 Great Recession, a strategy that the Federal Reserve pursued was the introduction of remedies such as mortgage relief or bailing out the banks to encourage people to take high-risk loans (Sheiner 2020). This is not the case for COVID-19 since the uncertainty associated with the effects of this virus is still undecided. Table 1 lists some facts comparing the 2009 Great Recession and COVID-19 (Hansen 2020).

**Table 1:** Quick facts about The 2009 Great Recession and COVID-19

The 2009 Great Recession	COVID-19
800,000 jobs were eliminated in March 2009; about 8.6 million jobs were lost in total	More than 20 million jobs were eliminated in April 2020, and more than 33 million jobs have been lost since the beginning of the pandemic
The highest unemployment rate was 10% in October 2009;	The unemployment rate for April 2020 reached 14.7%
The stock market lost about 40% off its peak value	The S&P 500 was down just under 14% from its peak in February 2020

Source: Hansen (2020)

Policy-makers need to compare and contrast the consequences of these sudden and devastating events and thus adapt their public policies to the lessons we have learned from similar past cases. It is also instructive for researchers and civilians to know how federal authorities have responded to these cases. The public announcements made by authorities and official statements, such as those published by the Federal Open Market Committee (FOMC), often include interesting but latent information that can be informative for research purposes. Using appropriate natural language processing (NLP) techniques, such statements can be mapped out to quantitative metrics and thus enable us to assess how effectively the authorities respond to events.

This paper introduces an NLP-based approach to compare and contrast chronological textual data (e.g., FOMC statements) and measures major events’ similarities to have a better insight into them. We compared the Federal Reserve statements published during the 2009 Great Recession with those published during the COVID-19 pandemic. The results of the document embedding technique Doc2Vec reveal around 10.8% increase in the dissimilarity of statements issued during the Great Recession and the COVID-19 pandemic. This indicates that the economic impacts of the COVID-19 pandemic might be even more severe than the consequences of the Great Recession on financial systems and banking.

Our research aims at answering the following research questions (RQ):

- RQ1: are the document embedding techniques such as Doc2Vec suitable for calculating representations of the FOMC post-meeting statements so that they can be used for downstream NLP tasks?
- RQ2: can disruptive events, such as the Great Recession and the COVID-19 pandemic, be portrayed in the embeddings? If so, how comparable are the similarity trends during the periods of these disruptive events?

We conducted two major experiments to answer the RQs mentioned above:

1. We calculated the similarities of consecutive statements using document embeddings as their representations. We captured the similarities over time during the Great Recession and the COVID-19 pandemic and visually analyzed them to investigate whether any similarities are observed during disruptive events.
2. We performed a clustering exercise of statements trying different clustering algorithms. The purpose of this is to explore the usability of the Doc2Vec representations of the statements in more complex tasks.

The key contributions of this paper are:

- It explores the use of Doc2Vec for generating vector representations of the FOMC post-meeting statements,
- It performs a similarity analysis of consecutive statements over time using TF-IDF representations and Doc2Vec embeddings, a more complex technique for generating document representations, as features,
- It performs a clustering exercise of statements to explore the use of Doc2Vec embeddings in a setting more complex than evaluating consecutive statements, providing evidence that more complex NLP techniques can be suitable for research using the FOMC post-meeting statements as the dataset,
- It explores how FOMC statements portray disruptive events and the way it affects their similarities.

The rest of this paper is organized as follows: (“Related work”) reviews the related work. In (“Methodology and technical background”), we provide the technical background of the NLP methods applied in this work. (“An analysis of federal reserve statements”) presents the experimental setup of the study. We report the results of the cosine similarities experiments for each period in (“Cosine similarity: the Great Recession vs. COVID-19 pandemic”). (“Cluster analysis of statements during the Great Recession”) presents the results of a cluster analysis performed on FOMC statements for the Great Recession. (“Conclusions and future work”) concludes the paper and sheds some light on future research directions.

## Related work

We organize the related work relevant to our study from 3 different perspectives: (1) how the FOMC post-meeting statements have been used by researchers using NLP techniques, (2) how researchers have studied the consequences of the COVID-19 pandemic and how these consequences can be portrayed in changes of the FOMC post-meeting statements, and (3) the embedding techniques applied to economics and social sciences in general.

### FOMC post-meeting statements and the NLP community

In February 1994, the FOMC started releasing its decisions on the federal funds rate target (Blinder et al. 2008). Soon after, in May 1999, the FOMC started publishing an assessment of its “bias” concerning future changes in monetary policy in its FOMC’s post-meeting statements. Three years later, FOMC began releasing votes attached with information related to voters’ names immediately after each meeting. In February 2005, the FOMC started to expedite releasing its minutes to make them available before the next FOMC meeting. In November 2007, the FOMC increased the frequency and expanded the content of its minutes (Blinder et al. 2008). The more recent FOMC’s post-meeting statements are now longer and more informative, taking the attention of NLP researchers. As a result, many research studies have focused on analyzing the FOMC’s post-meeting statements. This section briefly reviews the state-of-the-art NLP-based approaches to semantic analysis of the FOMC’s post-meeting statements.

In their survey paper, Mittermayer and Knolmayer (2006) reviewed numerous natural language processing tools for finance. The authors investigated text mining approaches in the form of knowledge detection and extraction. Following their definition of text-mining, they grouped several papers for analyzing news articles as well as FOMC statements, such as text preprocessing (Lewis 1992; Weiss et al. 2010), automated language detection of texts (Sullivan 2001), summarizing or abstracting texts (Gerstl et al. 2001), automated text categorization (Weiss et al. 2010), text clustering ((Weiss et al. 2010; Jain et al. 1999), extended information retrieval (Ferber 2003), and visualization of texts (Lin et al. 1991).

Several empirical works have investigated the FOMC communications and meeting notes. In particular, Kohn et al. (2003) and Gurkaynak et al. (2004) leveraged the FOMC communications such as the central bank communication’s effect on financial market volatility. These studies provide some evidence that the statements in FOMC meetings affect financial market variables.

Bernanke et al. (2004) evaluated the phrase of “considerable period” into the FOMC statement using the methods of modern empirical finance. The authors showed that after introducing the phrase into the FOMC statements, the sensitivity of 10 year treasury yields to news related to non-farm payroll employment increased.

Boukus and Rosenberg (2006) used Latent Semantic Analysis (LSA) to extract themes from FOMC meeting minutes. It has been reported that the prevalence of extracted themes affects treasury yields. Zadeh and Zollmann (2009) observed that the Federal Reserve Board meeting minutes could be used to predict volatility in stock market prices. They first harvested all the meeting minutes from online Web resources. Then, the collected meeting minutes were split into individual units. Finally, sentences were tokenized to predict volatility in the stock market.

Tang (2017) presents two supervised methods to measure the extent of discussion about specific researcher-defined topics in FOMC communications. The author uses a measure based on the frequency of a manually chosen set of terms defined to be specific to the topic of labor. The same approach has also been used for FOMC ((Frankel 2010; Husted et al. 2019; Cieslak and Vissing-Jorgensen 2021). The second measure used by Tang (2017) is based on training texts to measure investors’ sentiment from stock market message boards, a work similar to Antweiler and Franz (2004), and media slant of newspapers ((Gentzkow and Shapiro 2010). In addition, Latent Dirichlet Allocation (LDA) is used by Hansen et al. (2018) to estimate topics in FOMC transcripts. More specifically, their goal is to investigate the concentration of topics discussed during FOMC meetings on the publication of FOMC transcripts. In the same line, Gutierrez et al. (2020) explored how the estimated topic mixtures of FOMC statements during the Great Recession compare to those of the statements issued during the COVID-19 pandemic, and viceversa.

Acosta and Meade (2015) analyzed FOMC’s post-meeting statements using NLP-based techniques. The authors observe that the similarity of FOMC statements has been increased, and the contents have been changed slightly. This is the study from which the idea of comparing consecutive statements originated; hence, we adopt the idea of using the TF-IDF representation as a baseline for the comparison against a more sophisticated NLP technique (i.e., Doc2Vec). Note that we extend the idea of Acosta and Meade (2015) to explore the similarities of consecutive statements around the period of two important disruptive events while using a more sophisticated technique widely adopted by the NLP community.

Doh et al. (2020) utilize more sophisticated NLP techniques to calculate a novel measure of monetary policy stance that aims to include the tone with which the FOMC statements were emitted. Doh et al. propose a Universal Sentence Encoding architecture powered by a deep neural network to carry out their calculations.

### Analyses of FOMC statements during the COVID-19 pandemic

Bell and Blanchflower (2020) compare the US and UK labor markets before and during the COVID-19 outbreak by analyzing Federal Reserve’s reports. The authors compare the labor market performance in the US and UK and track the changes that have occurred monthly and daily since the beginning of March 2020. They show that during the COVID-19 pandemic, the labor market collapsed twenty times faster and much deeper than the Great Recession in 2008–2009. The authors observe that the unemployment rate due to the COVID-19 crisis in the US was around 20% in April 2020. However, the authors claim that the unemployment rate due to the COVID-19 crisis in the UK is hard to find given the lack of data, but it also has increased significantly.

Nicola et al. (2020) summarize the socioeconomic effects of COVID-19 on the world’s economy. The authors mention that social distancing, quarantine time, enforced border shutdowns, and restrictions on travel have led to an economic crisis and caused many jobs to be lost. According to Smialek (2020), the US Federal Reserve has decreased the interest rate by 0*.*5% to relieve the blow of the COVID-19 outbreak on the US Economy. FOMC, on March 23rd, 2020, announced a $300 billion lending program for Main Street businesses, as well as restarting the 2008–2009 Asset-Backed Loan facility (Elliott 2020).

On March 27th, 2020, the Trump administration supported the economy due to the COVID-19 crisis by offering a $2 trillion ‘virus-aid package’ called the CARES Act (Routley 2020). Sohrabi et al. (2020) investigated the extent of the outbreak with the World Health Organization (WHO) and showed that the COVID-19 outbreak was considered a global emergency on January 30th, 2020.

To the best of our knowledge, our work is the first study where the behavior of the similarities of consecutive FOMC post-meeting statements around disruptive events is analyzed with an NLP-based approach. Should the adoption of advanced NLP techniques be successful in the FOMC post-meeting statements, it would present a basis for researchers from economics and computer science interested in these types of data analysis.

### Embedding techniques in economics and social sciences

Beyond the use-case of FOMC statements, word embeddings are used as a real-valued vector for words for text analysis and big data in natural language processing. In the word embeddings approach, each word is represented as a d-dimensional word vector, capturing multiple different degrees of similarity between words and the relationships using this algorithm (Mikolov et al. 2013a). Several papers have been written about the applications of word embeddings in the political areas. The momentum of the embeddings techniques in these non-conventional areas is one of the motivators of our work. Because of this, we briefly review some of this research work on the use of embeddings in a broader range of subjects.

Rozado and Al-Gharbi (2021) use word embeddings models to measure political sentiment associations in news media content in the United States. They trained embedding models for each type of media content to calculate content-specific hidden associations between sentiment polarity (i.e., positive or negative) and terminology weighted with political concerns, such as descriptions of political affiliation and influential politicians, among others. They observed that the content for both left and right political orientations presents a positive polarity towards subjects and members of their ideology, whereas elements from the opposite side are negatively weighted; this correlates strongly (*r* > 0*.*7) with human ratings. Furthermore, their findings suggest that this political sentiment polarization has been increasing over time. Although the authors faced challenges when interpreting more complex sentiment associations, this study presents evidence of embeddings being able to capture ideological bias. Furthermore, Barbaglia et al. (2021) leverage the vast amount of news related to financial agents and expectations to train domain-specific word embeddings, with the purpose of forecasting the S&P500 index using several language model approaches from the state-of-the-art (e.g., DeepAR, a probabilistic auto-regressive recurrent neural network).

Word embeddings and topic models as computational textual analysis methods are used to describe Russian politics ideational dimensional (Indukaev 2021). These techniques develop a research design based on the specificities of the research question and available data. The study of Indukaev (2021) describes how the technological innovations and economic development in 2008–2012 changed when Putin replaced Medvedev and selected digitalization as a critical priority by discarding the modernization agenda. This study uses Instagram as the most extensive database in Russian media. The research strategy is to assemble the corpus based on technological and economic development, innovation, and digitalization, not including modernization and political change. This study uses word embeddings and topic models using the Text2Vec library for R with 50 topics and then runs models with 45 and 55 topics. The results show that modernization before January 1st, 2012 was close to political liberalization, but innovation became less associated with political change. The results show that the digitization program was not related to political liberalization Indukaev (2021).

The study of Liu et al. (2021) analyzes the factors contributing to political polarization in the US by proposing a framework for depolarization news articles. The framework discovers polar language and replaces it with neutral expressions. Using data from the media cloud, an attribute-aware word embeddings model identifies the polar words. Then a text annealing depolarization algorithm (TADA) is proposed to create text generation by retrieving neutral expressions from the word embedding model without decreasing ideological polarity. The framework is evaluated by comparing the depolarized output of models in fully and semi-automatic modes. The results show that over 90.1 and 78.3% of paragraphs were successfully depolarized in semi- and fully automatic modes, respectively. In comparing the original and depolarized texts, over 81.2% of the testers agree that the non-polar content information is well preserved, and 79% agree that depolarization does not harm semantic correctness. Because of the limitations of memory, the proposed framework cannot handle a large number of attributes. Then, the framework can be extended by employing contextual language models and considering more memory-efficient models for creating multi-attribute-aware embeddings and exploring continuous attributes.

In another study, Chen et al. (2020) collected a multilingual COVID-19 twitter dataset starting from January 28th, 2020. They showed how Twitter responds to coronavirus-related events with social distancing measures, travel bans, among others. Sciandra (2020) analyzed the media communication about the COVID-19 crisis in Italy by using the Twitter dataset before and through the national lockdown. First, a bag-of-words (BoW) approach was used. Sentiment analysis was performed by considering each tweet and the sentiment content of the whole tweet. The sign function was applied to the sum of the sentiment content of words. The NRC and the TextWiller lexicons were selected to extract a sentiment score for each tweet. Then, three different measures were selected to assess the level of overlap between the two sentient distributions. The results showed that 48% of tweets had been in the exact sentiment of two lexicons. The values of Cohen’s Kappa and W coefficients were significant and equal to 1/3 and 2/3 of the theoretical maximum, respectively. Since a BoW approach could not capture the semantics and context similarities, word embedding techniques were used. The results show that word embedding could not present a higher predictive ability than the frequency vectors of terms. Although we do not perform prediction in this work, it is worth noting that the adoption of a more sophisticated vector representation did not yield improvements in the study’s metrics. This contrasts with our findings when detecting disruptive events using document embeddings using such sophisticated techniques.

Word embeddings augmented models with the political metadata and trained on large-scale parliamentary corpora were fitted in another study (Rheault and Cochrane 2020) for Britain, Canada, and the United States. The results were evaluated using different measures and showed that the word embeddings models successfully capture latent concepts in political Language studies.

Several efforts have gone to detect and solve the problem of bias in textual data using machine learning and NLP techniques. Bolukbasi et al. (2016) developed a new method for quantifying direct and indirect biases arising from a corpus of text and developing algorithms to debias the embeddings by using crowd-worker evaluation and standard benchmarks. In another study, Gordon et al. (2020) used the same methods for political bias. Unlike the previous study (Bolukbasi et al. 2016), Gordon et al. (2020) developed a methodology to calculate a political bias subspace which can be used for computing other types of biases. A collection of a 26 GB corpus of tweets from two political parties in the US shows more political bias in the tweets from both parties than tweets from the politicization of the same party. Political bias is modeled as a binary choice along one axis.

In another study, Jenkins and Owen (2020) refer to a political bias in the media contents, which influences the public opinions in South Korea. In this study, a data-driven approach was developed to compute the political bias media contents using Doc2Vec and social data from Facebook. Jenkins and Owen (2020) proposed a novel paragraph embeddings method for learning representations of the financial earning calls, which are essential in trading strategies and knowing economic trends. This study collected quarterly earning call data from Refinitiv via Thomson Reuters, and 10,000 call transcripts about 615,603 unique text documents or paragraphs were analyzed. The proposed model was compared with the other embedding methods such as LDA and Doc2Vec in terms of classification accuracy, area under the curve (AUC), receiver operating characteristics (ROC), and average precision (AP). The results show an increase in accuracy, AUC (ROC), and AP in the proposed model than the other methods. The proposed model also provides a strong sense of document-to-document and word-to-document similarity.

In this paper, we leverage NLP approaches to evaluate the similarity of consecutive FOMC post-meeting statements issued during the Great Recession and the COVID-19 pandemic. More specifically, we applied Doc2Vec to generate document embeddings for the statements, in addition to obtaining their TF-IDF representation as was initially proposed by Acosta and Meade (2015), which we used as a baseline. A detailed description of our experiments is presented in (“An analysis of federal reserve statements”).

## Methodology and technical background

This section presents the methodology used in our work alongside the details of the techniques used to carry out our experiments. We conducted two major experiments:

## Similarity analysis of consecutive statements

**Fig. 1:**
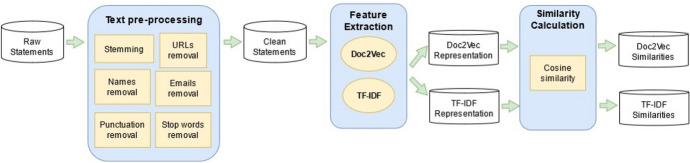
Flowchart showing the methodology applied for the generation of statements’ similarities. We applied widely used text preprocessing procedures on the raw statements. Next, we performed feature extraction using both TF-IDF and Doc2Vec. Once we obtained both document representations, we calculated the cosine similarities between each statement issued at timestamp t and the one released at the previous timestamp t − 1

We calculated the pairwise similarities between consecutive statements and analyzed them over time. Figure 1 shows the approach we used to perform this task. We divided this Flowchart into three main blocks: (a) data preprocessing, (b) feature extraction, and (c) similarity calculation.

- Text preprocessing: given the initial set of raw statements, we applied standard textual preprocessing techniques such as the removal of URLs, stop words (i.e., words that serve a syntactic purpose rather than giving content to the sentence), punctuation, email addresses, stemming (i.e., extracting the roots of the words), and names. The latter is required as the statements contain several names of authorities from the Federal Reserve.
- Feature extraction: once the statements are processed, a feature extraction step is needed to generate a document representation that can be used to calculate similarities between statements. In our work, we apply the TF-IDF and Doc2Vec techniques to perform feature extraction. Both methods have different principles as a basis; TF-IDF relies on the post-processing of raw counts of term frequencies, whereas Doc2Vec aims to capture more complex semantics.
- Similarity calculation: after the feature extraction step is complete, each statement is assigned a vector (i.e., the TF-IDF or Doc2Vec representation). Using these vectors is possible to calculate pairwise similarities between consecutive statements. In this work, we used a standard similarity metric in natural language processing: the cosine similarity. Next, these similarities were plotted over time to examine its behavior. Since we compared the similarities during two different periods, the Great Recession and the COVID-19 pandemic, we performed the analysis using two datasets of statements.

## Clustering of statements issued during the Great Recession

**Fig. 2:**
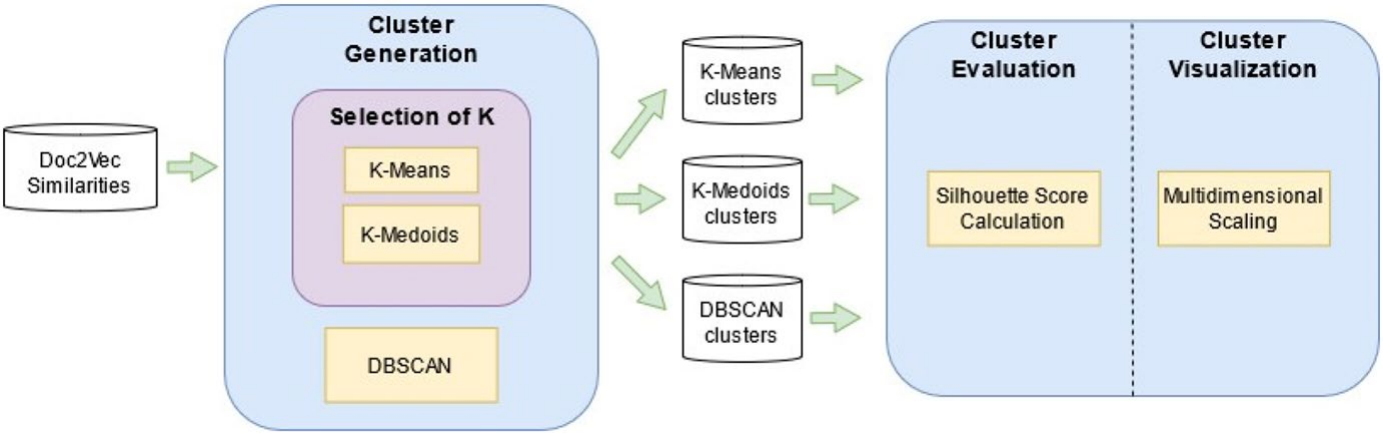
Flowchart of the clustering of statements issued during the Great Recession. The input for this task is the complete set pairwise cosine similarities of statements. Next, the cluster generation phase was performed using K-means, K-medoids, and DBSCAN. In the case of K-means and K-medoids, we selected the optimum value for *K*. After the clusters were generated for each algorithm, we evaluated them using the silhouette score and visualized a 2-dimensional projection of them through multidimensional scaling

Our justification to carry out this task is not as straightforward as that of our first task: consider a clustering scheme of statements that allows a reasonable interpretation (e.g., the clusters obtained aligned with the socioeconomic landscape), this suggests that the underlying vector representation of the statements can capture the semantics of the documents successfully; hence, the Doc2Vec representations of the statements could be used for several standard downstream tasks in natural language processing. Figure 2 depicts our approach to carry out this task. The Flowchart is divided into three sections: (a) cluster generation, (b) cluster evaluation, and (c) cluster visualization.

- Cluster generation: a fundamental step in clustering is to calculate the pairwise distances between the objects to be clustered. In our work, we performed this extending the Similarity calculation step to all pairs of statements instead of only consecutive ones. We utilized three clustering algorithms: K-Means, K-Medoids, and DBSCAN. Since K-means and K-medoids are partition-based clustering algorithms, we applied an extra step for these methods:
  Selection of K: K-means and K-medoids partition the universe of objects into K partitions (further details of these algorithms are given in (“K-means and K-medoids”), where K must be set beforehand. To select the best value of K for our problem, we conducted an optimization process based on elbow graphs.
  The output of this phase is a data structure that maps statements to clusters generated for the three algorithms.
- Cluster evaluation: we systematically evaluated the clusters obtained in the past phase. We used the silhouette score (Rousseeuw 1987) as the criterion to achieve this.
- Cluster visualization: an immediate visual inspection of the clusters is not possible because of the high-dimensionality of the representations of the statements. To address this matter, we employed multidimensional scaling to calculate bi-dimensional projections of the clusters and aid the interpretation of results. We used this technique to preserve the Euclidean distances between samples when performing the projection, in contrast to other widely adopted projection techniques (e.g., TSNE).

Due to data availability, we only utilized statements from our first dataset (i.e., the Great Recession) in this task.

Next, we give a brief technical description of each algorithm and method used in our experiments.

### Cosine (dis)similarity

The cosine similarity between two vectors, *x* and *y,* is

$${\text{Cos}} Sim\left( {x,y} \right) = \frac{{x^{T} y}}{\left\| x \right\|\left\| y \right\|}$$

Cosine similarity ranges from − 1 to 1. Vectors whose cosine similarity is closer to − 1 are considered dissimilar, while they are considered similar if the value is closer to 1. In this work, we applied cosine similarity directly when comparing our results with that of different FOMC statements. However, an important part of our work is visualizing high-dimensional data using MDS, which requires dissimilarity between samples. For this purpose, we utilized the cosine dissimilarity, defined as *CosDissim*(*x,y*) = 1 − *CosSim*(*x,y*) for vectors *x* and *y*.

### TF-IDF

The purpose of the Term Frequency-Inverse Document Frequency (TF-IDF) processing of a document-term frequency matrix is to weigh the terms so that rare terms over documents hold higher values, and common ones have lower values. The idea behind this approach is that rare words are more capable of distinguishing documents than common words.

Let *A* be a *d* ✕ *t* document-term frequency matrix, where *d* is the number of documents in our dataset and *t* is the number of unique terms obtained in the text. Each entry ${A}_{i,j}$ contains the frequency of the term ${t}_{j}$ in the document ${d}_{i}$. The TF-IDF processing generates a new matrix $T$ where each entry is computed as follows:

$${T}_{i,j}={A}_{i,j}\cdot idf\left(j\right),$$

The factor $\mathrm{idf}\left(\mathrm{j}\right)$ is calculated as

$$idf\left(j\right)=\mathrm{log}\left(\frac{d}{{f}_{j}}\right),$$

where ${\mathrm{f}}_{\mathrm{j}}$ is the number of documents in which the term ${t}_{j}$ is present.

### Doc2Vec

Doc2Vec (Le et al. 2014) is a neural network-based document embedding technique that aims to generate document vectors similar to what Word2Vec (Mikolov et al. 2013b) does with word vectors. Like Word2Vec, Doc2Vec works under the Distributional Hypothesis (i.e., words used in similar contexts share similar semantics) to set the objective function during the training phase of the model. Consequently, the framework for training document vectors is very similar to that of word vectors.

Regarding dimensionality, Doc2Vec performs feature extraction generating *p*-dimensional dense document vectors using variable-length documents as input. As *p* is an integer considerably smaller than the number of dimensions of frequency-based embeddings, Doc2Vec is usually utilized to generate features fed as input to other machine learning methods that benefit from the low-dimensional features.

Note that, unlike TF-IDF, Doc2Vec generates dense document embeddings trained under the Distributional Hypothesis, so it takes into account the context within the documents.

### K-means and K-medoids

*K*-means is a well-known technique for partitioning a set of objects into *k* clusters, where *k* is known beforehand. A centroid characterizes each cluster, which corresponds to the average of its assigned samples. Formally, the optimum cluster assignment minimizes the objective function

$$J =\sum_{j=1}^{k}\sum_{i=1}^{N}dist\left({x}_{i}^{\left(j\right)},{c}_{j}\right)$$

where *dist *(*x*_*i*_*,c*_*j*_) is the Euclidean distance between the sample *x*_*i*_ and its assigned cluster’s centroid *c*_*j*_ for *N* objects and *k* clusters. In contrast, K-medoids aims to minimize the same objective function while using medoids instead of centroids for each cluster, where a medoid is the most representative object in a cluster. This constitutes the essential difference between K-means and K-medoids: centers of clusters are always part of the K-medoids dataset, while they are not for K-means.

### DBSCAN

Density-Based Spatial Clustering of Applications with Noise (DBSCAN) is a density-based spatial clustering technique that can detect clusters of different sizes and shapes. Clusters are defined as samples positioned in a connected region of the space where samples are densely agglomerated. Samples are considered to be part of a cluster if the density is above a threshold. All samples that are not considered to be part of a cluster are deemed as noise. Two crucial hyperparameters in this algorithm are *minPts* and ε. *minPts* denotes the minimum number of points in a sphere of radius, which is the threshold mentioned above, whereas ε is the maximum distance between two objects for one to be considered to be part of the neighborhood of the other.

### Silhouette scores for optimal clustering

Cluster silhouettes (Rousseeuw 1987) are a method for visualizing and assessing the quality of the clusters obtained after applying a clustering procedure. Intuitively, each cluster *A* is characterized by a *silhouette*, which is determined considering the compactness of *A* and its separability to the rest of the clusters. Each sample *i* in a dataset has its silhouette score (i.e., *s*(*i*)), determined by

$$J = \sum\limits_{j = 1}^{k} {\sum\limits_{i = 1}^{N} {dist\left( {x_{i}^{\left( j \right)} ,c_{j} } \right)} }$$

where *a*(*i*) is the mean intra-cluster dissimilarity for the cluster in which sample *i* is contained, and *b*(*i*) is the dissimilarity measure between the sample *i* and its closest cluster that is not the cluster in which it is contained. Note that − 1 ≤ *s*(*i*) ≤ 1 where samples with silhouette scores close to 1 suggest a correct cluster assignment; whereas, values close to − 1 suggest that the sample was incorrectly assigned to the cluster.

### Multidimensional scaling (MDS)

Multidimensional Scaling (MDS) is a technique that monotonically maps dissimilarities of samples in a potentially high dimensional space to a low-dimensional embedding, so that the distances between samples in the low-dimensional space are in concordance with the dissimilarities in the original space (i.e., two samples that are dissimilar are far apart in the embedding) (Borg and Groenen 2005).

## An analysis of federal reserve statements

We present the experimental setup and the analysis results performed on a set of Federal Reserve statements to compare the Federal Reserve’s statements output in the Great Recession and during the COVID-19 pandemic.

### Experimental setup

We implemented both K-means and K-medoids techniques in our analysis because K-means is more sensitive to outliers than K-medoids, as very far apart objects can heavily contribute to a centroid, moving it towards that direction. In this work, we used Euclidean and cosine distances to calculate the silhouette scores. We used these distances for the clusters obtained using K-means and K-medoids, respectively. We embedded Federal Reserve statements using Doc2Vec, where the *p*-dimensional vector was set to *p* = 20.

We used MDS to perform dimensionality reduction to visualize clusters. In addition, we used both Euclidean distance and cosine dissimilarity when generating the embeddings for the results of K-means and K-medoids, respectively. In our experiments, we set *minPts* = 3 and ε = 0.1, these values were obtained empirically after trying different ranges for *minPts* and ε after getting results that did not differ significantly from each other, showing that for this dataset and configuration, the selection of hyperparameters for DBSCAN is not an important source of sensitivity.

### Dataset

Our first dataset consists of 106 FOMC’s post-meeting statements. The first one is the statement issued in February 2005, and the last one is the statement issued in March 2018. To carry out our experiments and make our results comparable with Acosta and Meade (2015), we restricted our dataset to the statements issued from February 2005 to October 2014. After applying this criterion, our final dataset consisted of 79 statements.

The second dataset, used for exploring the impact of the COVID-19 outbreak in the post-meeting statements, consists of statements issued from September 2019 to July 2020. During March 2020, the frequency of statements slightly increased; because of this, there are ten statements in this dataset.

Note that we utilized the statements as a whole in our analysis. Although we acknowledge that there is a possibility to conduct our experiments in a more fine-grained manner, such as considering the paragraphs as the minimum units for the similarity comparison, the effort to track effectively the relevant paragraphs throughout the statements (e.g., delimitate the paragraphs that refer to the economic outlook using a labeling procedure) would be massive. Furthermore, the internal structure of the statements presents abrupt changes when the COVID-19 pandemic started being a concern for the Federal Reserve. Because of this, we limit our analysis to the statements as atomic units.

For both datasets, we excluded the voting information as in Acosta and Meade (2015). Additionally, we also excluded the section that refers explicitly to the decisions regarding monetary policy implementation when it is present.

### Implementation and libraries

We developed Python 3.8 scripts to conduct our experiments. We utilized the Gensim implementation (Rehurek and Sojka 2010)) of Doc2Vec that provides a Python interface. For implementing MDS, the silhouette scores, TF-IDF, and the clustering algorithms, we used the Scikit-learn library (Pedregosa et al. 2011). For stemming, we used the NLTK Python library (Loper and Bird 2002).

## Cosine similarity: the Great Recession vs. COVID-19 pandemic

### Cosine similarities of statements of the Great Recession

**Fig. 3:**
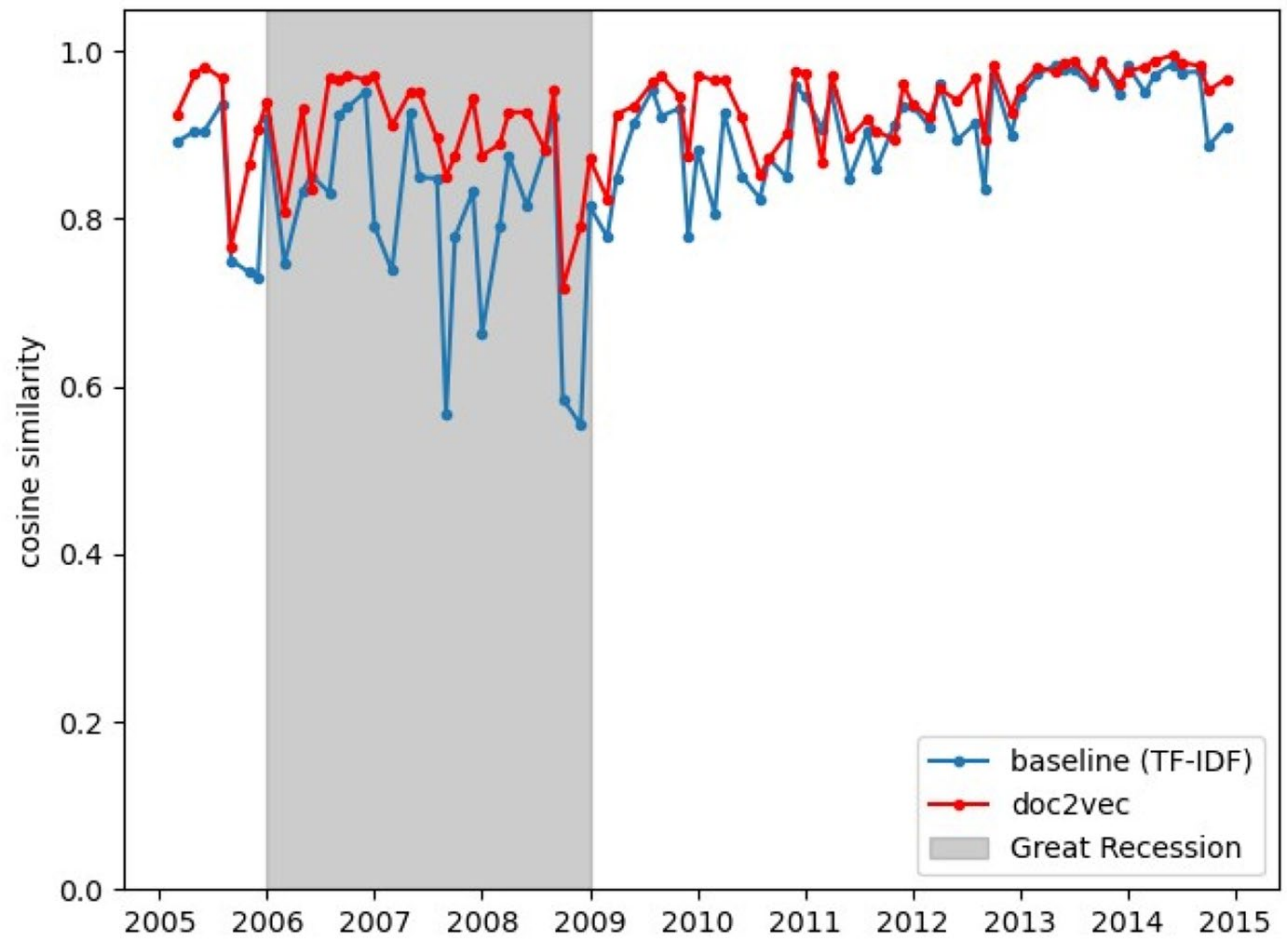
Cosine similarity of statements over the Great Recession period. The Great Recession is represented as the grey area. It is visible that the most critical similarity drops occurred during the period of the Great Recession

Figure 3 shows the cosine similarity of the Doc2Vec document embeddings. The *x*-axis is the chronological order of statements and their years; whereas, the *y*-axis is the cosine similarity between two consecutive statements. We calculated the cosine similarity of each document using the same procedure described by Acosta and Meade (2015) (i.e., each point in Fig. 3 is the cosine similarity between document *d*_*t*_ and document *d*_*t*−1_ for each timestamp *t*). Following the idea presented in Acosta and Meade (2015), we also implemented the TF-IDF features after preprocessing the statements using stemming. To have a better insight into the data, Table 2 shows descriptive statistics for the cosine similarities of the statements.

**Table 2:** Descriptive statistics of the cosine similarities

Crisis	Technique	Mean	Std	Min	Max
Great Recession	Doc2Vec	0.905	0.062	0.718	0.971
	Baseline	0.807	0.112	0.554	0.951
COVID-19	Doc2Vec	0.889	0.136	0.648	0.998
	Baseline	0.858	0.148	0.644	0.991

In the case of the Great Recession, we report the descriptive statistics of the statements issued only during this period

In Fig. 3. In the case of the Great Recession, and to address the difference in the size of the datasets, we limited the similarities in Fig. 3 to those of statements issued between the years 2006 and 2009.

Looking at Fig. 3, it is apparent that Doc2Vec reduces the volatility of cosine similarities between documents (i.e., Federal Reserve statements) in comparison to the baseline (i.e., TF-IDF). An intriguing observation is that the volatility reduction is performed without sacrificing the initial pattern of similarities; this could mean that the Doc2Vec representations can preserve the semantics of more sparse techniques (i.e., TF-IDF). This can be seen if we consider that the volatility of the cosine similarities for the baseline is approximately 0*.*0090; whereas, the volatility for the similarities of the Doc2Vec representation is approximately 0*.*0031. This value corresponds to 34% of the original (baseline) volatility. Hence, it seems Doc2Vec re-scales similarities metrics by compressing their dimensions. This has important implications when using machine learning techniques with the document embeddings as input features since the patterns of more sparse data can be captured within fewer dimensions. Since the Curse of Dimensionality is a well-identified issue in machine learning, this finding suggests a clear advantage in using FOMC statements embeddings in machine learning systems.

#### Analysis findings and the connection with 2008 financial crisis

Historically, the FOMC makes any changes, notably to the Federal Funds rate (interest rate) and the discount rate (the minimum interest rate for lending to other banks) in the official statements. The differences between the FOMC statements regularly refer to the monetary policy goals of the Federal Reserve in fostering economic conditions during the time. The general aim and policy of the Federal Reserve are to achieve sustainable economic growth and price stability by setting a response policy to economic adversity and crises.

Figure 3 highlights three significant changes that had occurred in the Federal Reserve statements during 2006–2009. During that period, the US economy experienced several banking failures during the financial crisis of 2007–2009. The Federal Reserve responded with a broad array of actions to the financial crisis and expanded the size of its balance sheet over time. The similarities trend for the baseline in Fig. 3 follows the same pattern as in Acosta and Meade (2015) (i.e., the drops in similarity occur around the exact dates). However, there are minor variations, probably due to the differences in the text preprocessing steps that we performed after replicating the idea of Acosta and Meade (2015).

According to Bernanke (2009), in June 2006, the Federal Reserve policy-makers boosted its target for the Federal Funds rate to 5*.*25 percent and the Discount rate to 6*.*25 percent, in the straight rate increase since 2004. In August 2006, the Federal Reserve kept rates stable because of the cooling of the housing markets and the lagged effects of the increase in interest rates and energy prices.

These factors and the economic slowdown caused the Federal Reserve policy-makers to keep rates unchanged in their official statements until August 2007. The collapse in the housing market in 2006 and 2007 had a substantial impact on the banking system. In August 2007, financial markets became volatile, credit conditions became tighter for some households and businesses, the downside risks to economic growth increased, and inflation failed to moderate.

Due to the downturn of the economic conditions, the Federal Reserve kept the Federal Funds rate at 5*.*25 percent and reduced the discount rate from 6*.*25 percent to 5*.*75 percent in August 2007. The spread between the primary credit report and the Federal Funds rate reduced to 50 basis points from the 100-point spread established in January 2002.

Sixteen months later, the FOMC lowered the target for the Federal Funds rate to nearly zero in response to the downturn of economic conditions. The aim was to help with easing money and credit. Many factors have explained the adoption of this policy which are: the weak outlook for economic activity, the low growth of consumer spending, uncertainty about the higher inflation, the lower demand for US exports, the low growth of industrial production, the high unemployment, stock downturn, and the failures of Wall Street firms Bear Stearns and Lehman Brothers in 2008.

The FOMC adopted a zero interest rate policy (ZIRP) during December 2008–December 2015, but the initial effects were for the 2007–2009 financial crisis. The Federal Reserve also reduced the discount rate for the same period. Furthermore, the Federal Reserve adopted Quantitative Easing (QE) monetary policy in several rounds.

### Cosine similarities of statements during COVID-19

**Fig. 4:**
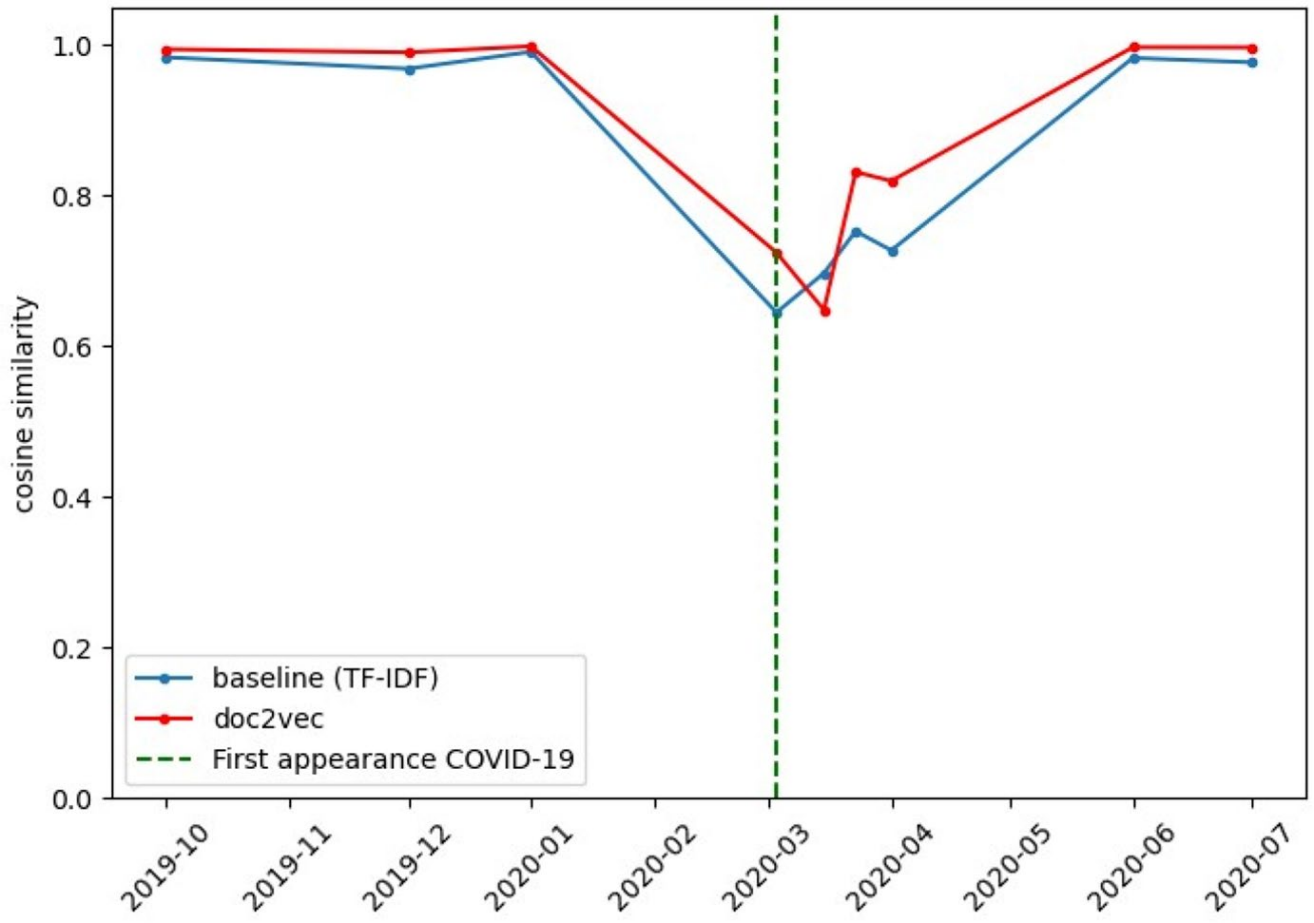
Cosine similarity of statements issued near to and during the COVID-19 outbreak. There was an evident drop in similarities when the COVID-19 pandemic first appeared (vertical green dashed line). This drop is present for both the baseline (TF-IDF) and our Doc2Vec representation

Figure 4 shows the cosine similarities for the statements issued from September 2019 to July 2020. The cosine similarities were calculated using the same procedure as those of (Fig. 3). As mentioned in Section 4, 10 statements were issued during this period.

Similar to what we did using our first dataset, we calculated the TF-IDF representation of statements using the same procedure described previously.

Table 2 also reports descriptive statistics of the cosine similarities of statements issued during the COVID-19 outbreak using Doc2Vec and the baseline method. Furthermore, Fig. 4 also shows the statement where the COVID-19 outbreak was explicitly addressed for the first time, which is the one issued on March 3rd, 2020.

#### Analysis findings and the connection with the COVID-19 pandemic

Typically, there are eight FOMC post-meeting statements during each year, however in less than a year (from September 2019 to July 2020), the FOMC issued 10 statements. Figure 4 shows that this increase in frequency is due to the statements issued in March 2020. Only this month, the FOMC released three post-meeting statements (more specifically, on March 3rd, 15th, and 23rd). Additionally, these statements report the most significant drops in cosine similarities of consecutive statements in (Fig. 4).

FOMC statements from September 2019 to January 2020 address topics, policies, and standard measures for these documents, as described in (“Analysis findings and the connection with 2008 financial crisis”). Nevertheless, the statement of March 3rd, 2020, presented an essential disruption in terms of extension and content concerning past statements, as it was the first to address the COVID-19 pandemic and how it posed an evolving risk to the economic activity. Moreover, this statement was considerably shorter than the immediate past one (88 words on March 3rd, 2020, compared to 275 words on January 2020). It discussed only the decrease of the target range for the Federal Funds rate as the immediate action to keep supporting maximum employment and price stability during the pandemic.

In March 2020, the FOMC post-meeting statement addressed the measures taken towards its goals of maximum employment and price stability in a broader way as in statements before March 2020. Nonetheless, the new pandemic-induced global financial conditions and the implications that public health might have for the economic outlook are also considered for the first time.

The statement issued on March 23rd, 2020, refers primarily to actions aiming to support the flow of credit households and businesses during the pandemic. Onward, the statements directly refer to the massive impact that the pandemic had on human wellness and the economy on both a global scale and in the United States, in addition to the full range of measures to support the achievement of the goals of the Federal Reserve.

The drop in the cosine similarity for March 3rd, 2020, in Fig. 4, agrees with the abrupt change of topics and content of the FOMC post-meeting statements due to the COVID-19 pandemic. The mainly increasing later similarities suggest that the statements started stabilizing in terms of content, which agrees with our findings when inspecting the documents, as it was described in the previous paragraphs.

There is an important common factor between the cosine similarities of statements issued during the Great Recession and those issued during and after the advent of COVID-19. From Table 2, the minimum cosine similarity in Fig. 3 for our implementation of Doc2Vec is 0.718; this value was reported during the Great Recession. Furthermore, the same phenomenon can be observed for the baseline values. On the other hand, Fig. 4 shows consistently high values for statement similarities until the appearance of COVID-19, when the similarity drops to 0.648 for Doc2Vec (see Table 2). In comparison, when considering Doc2Vec, the lowest cosine similarity during the Great Recession is 10.8% larger than that of the COVID-19 pandemic.

On the other hand, for TF-IDF, such similarity is 13.9% higher. The mismatch between these differences might be due to the way that each embedding technique works. For instance, Doc2Vec generates the document vector for a statement considering the vectors of each word contained in the document. In contrast, TF-IDF follows a frequency-based approach to create the embedding. Considering this, is reasonable to assume that the Doc2Vec method captures the semantic of a statement in a more integrated way than TF-IDF; hence, its values would be more representative of the content of the whole statement. This finding suggests that both the Doc2Vec and TF-IDF representations are suitable for detecting changes in similarities of consecutive statements, as disrupting events like the Great Recession and the COVID-19 pandemic are identifiable when analyzing the cosine similarities of statements issued during their respective periods.

## Cluster analysis of statements during the great recession

We also performed cluster analysis on the first dataset (i.e., the Great Recession dataset) to find out whether it is possible to cluster Federal Reserve statements and thus be able to classify them. We did not perform similar clustering for the COVID-19 dataset since there were only 10 statements in the dataset.

**Fig. 5:**
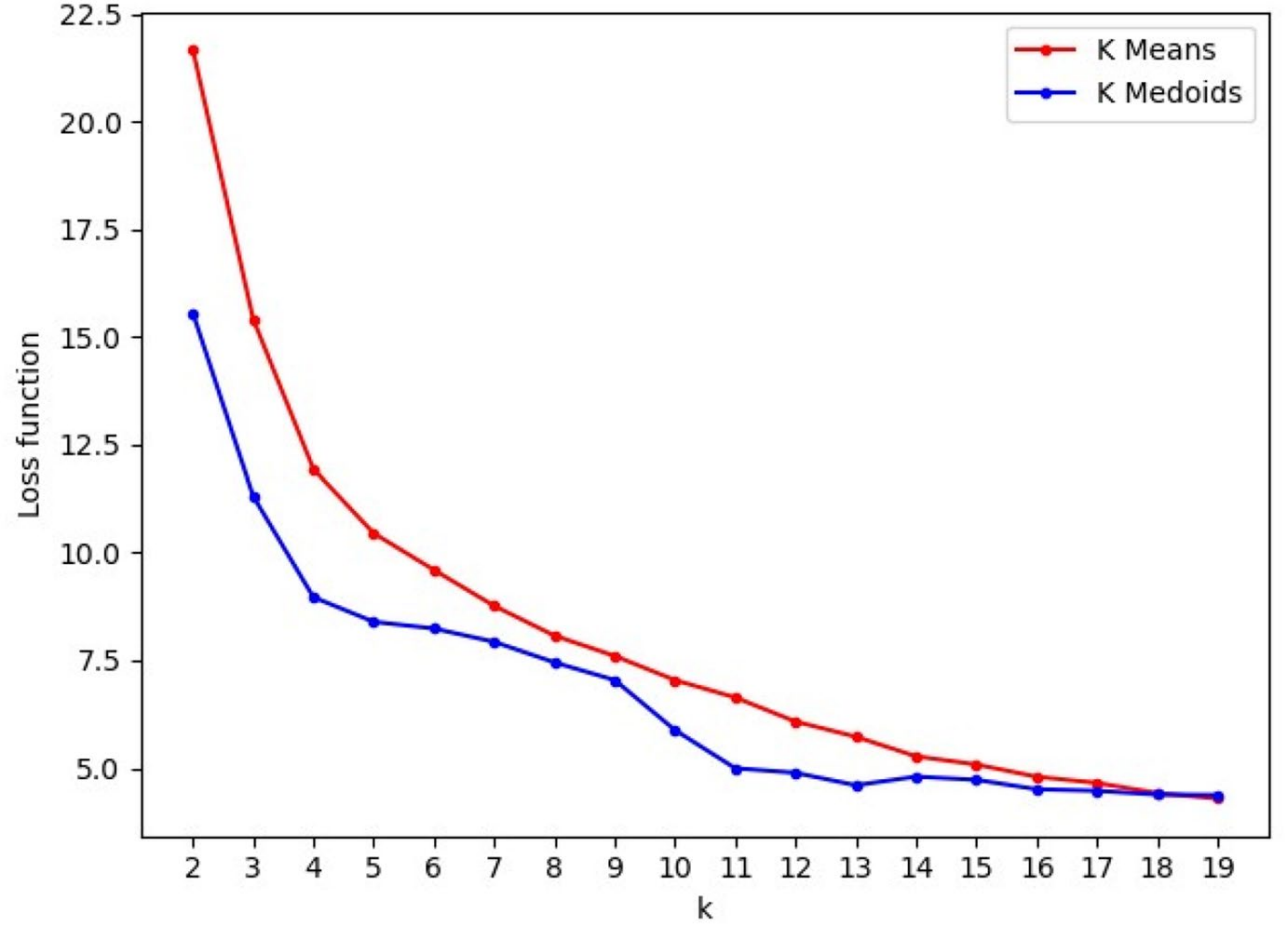
Elbow graph for K-means and K-medoids. The steepest decrease in the loss function occurs when transitioning from three to four clusters for both K-means and K-medoids; this suggests that *k* = 4 is the optimum number of clusters

As we do not know the optimal number of clusters (k) that best fits the document embeddings in our dataset, we generated elbow graphs to identify the best *k*, ranging from 2 up to 19. Figure 5 shows the elbow graphs for K-means and K-medoids where the *x*-axis represents various values for *k*; whereas, the *y*-axis shows the loss values obtained for each *k*. In the case of K-means, the loss function corresponds to the sum of the Euclidean distances between each sample and its assigned cluster’s centroid, while for K-medoids, it is the sum of their cosine dissimilarities.

Although the loss function is monotonically decreasing for K-means, the loss reduction curve is relatively smooth, so there is no precise elbow shape. As a result, it is not clear which optimal value *k* should be chosen using this graph. On the other side, Fig. 5 also indicates that the decreasing trend slows down significantly after *k* = 4 for K-medoids, which suggests that the optimum value exists at *k* = 4, in the case of K-medoids.

**Fig. 6:**
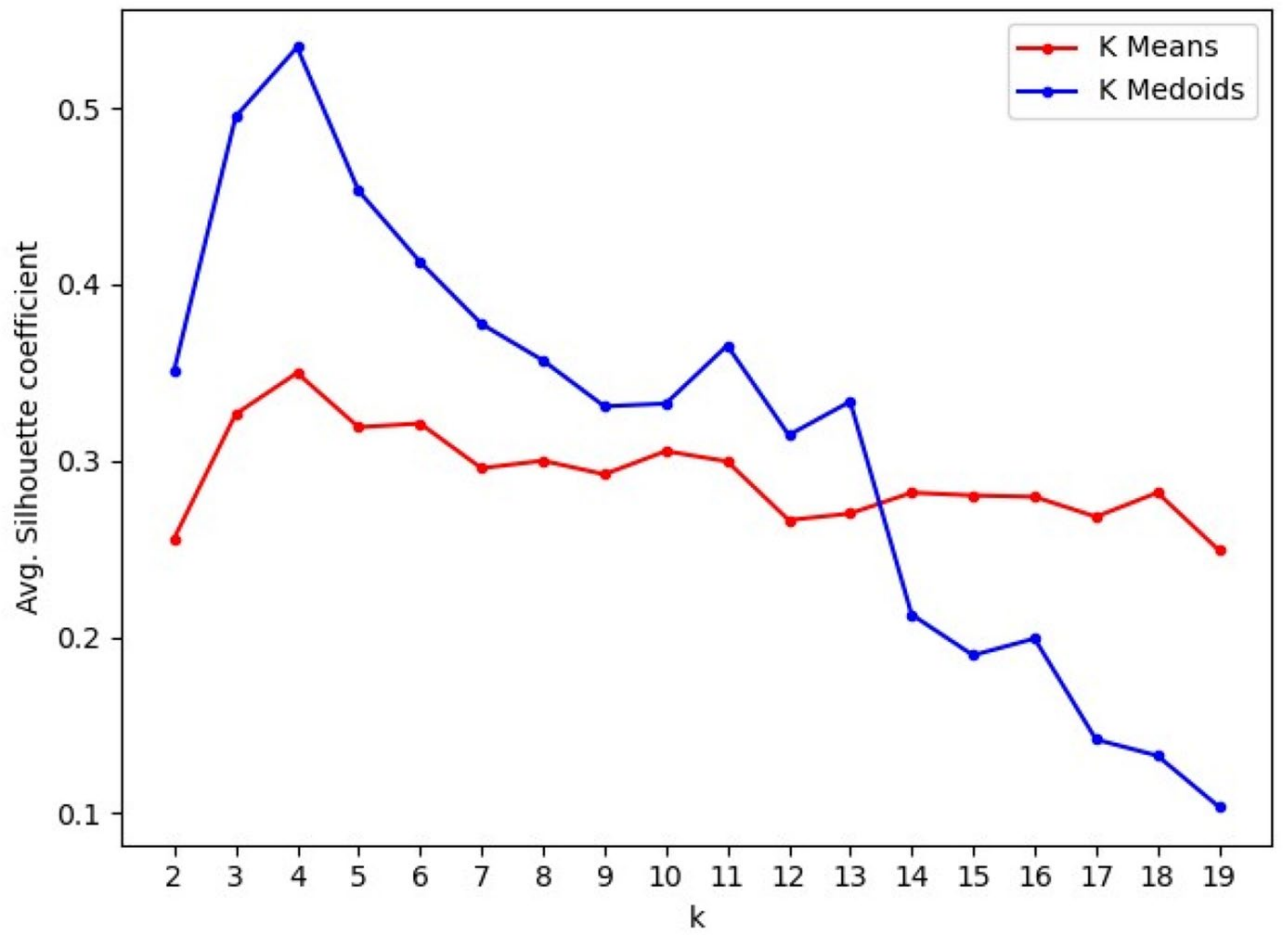
Average silhouette score per number of clusters (k). The highest average silhouette score is reached at *K* = 4 for both K-means and K-medoids

To add a method to select the optimal *k*, we calculated the average silhouette score for each *k* as suggested in Rousseeuw (1987). Figure 6 shows the average silhouette score per *k* for K-means and K-medoids. Using this approach, K-medoids generates consistently better clusters than K-means, as their scores are more significant for all *k* < 14. However, both clustering techniques report the highest silhouette score at *k* = 4 with 0*.*349 and 0*.*534 for K-means and K-medoids, respectively. As a result, we based our remaining analysis on clustering with *k* = 4.

### Cluster visualization

**Fig. 7:**
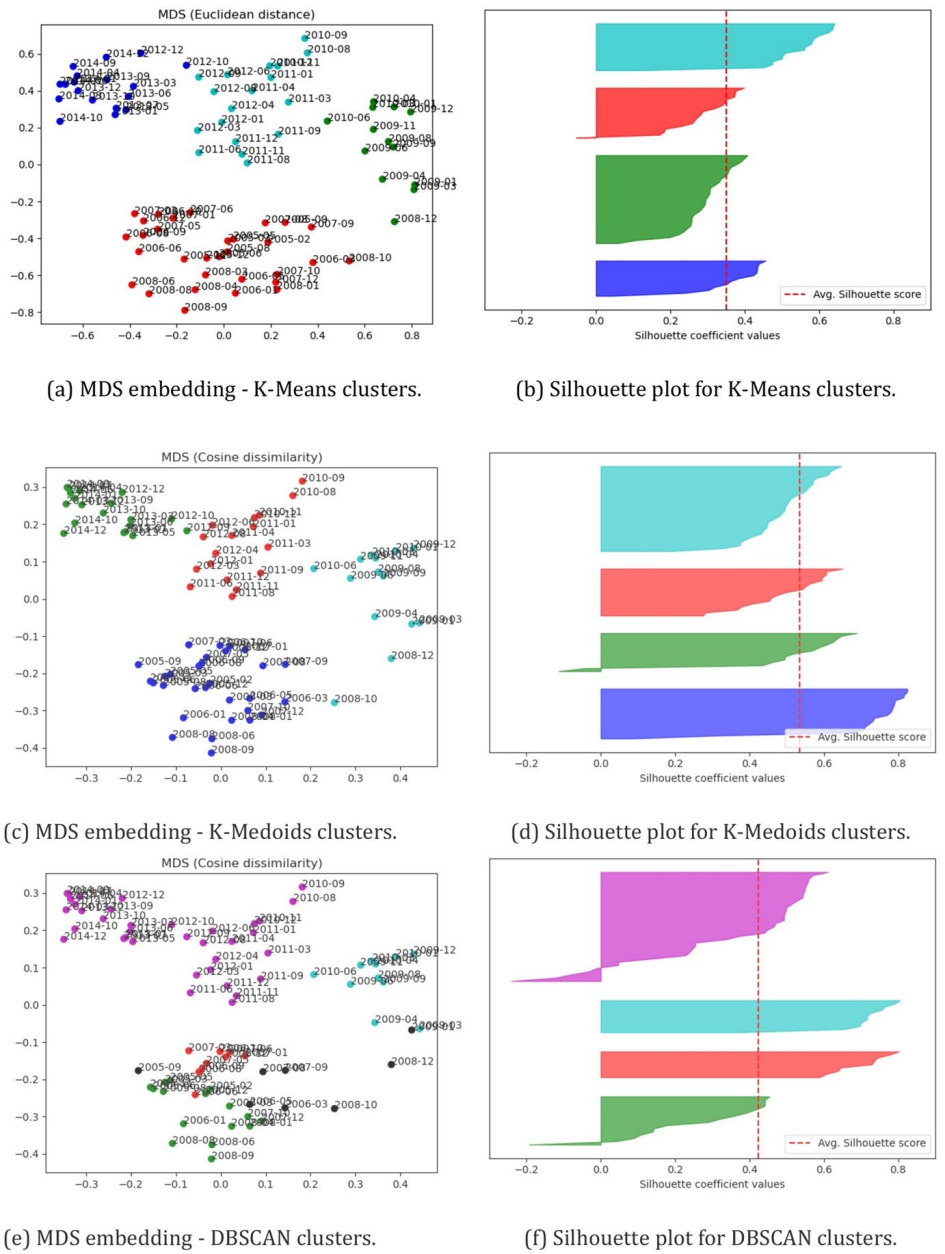
Cluster evaluation for K-means, K-medoids, and DBSCAN using silhouette scores (The Great Recession Period)

**Fig. 8:**
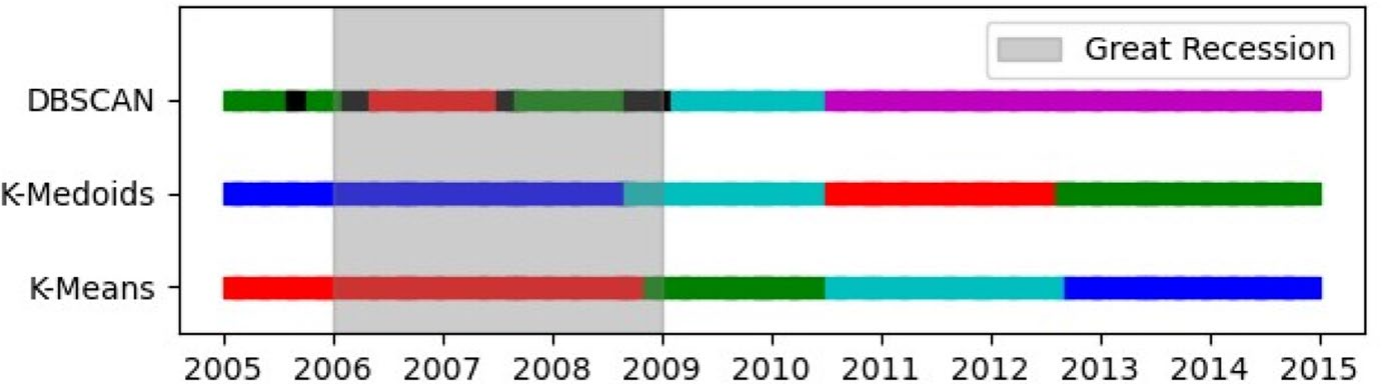
Clustering results of statements over time (the Great Recession). The Great Recession is represented as the grey area. It is visible that almost all clusters are aligned chronologically. Also, all statements deemed as noise by the DBSCAN clustering technique were issued during or around the Great Recession period

Figure 7a, c, e illustrate the MDS embedding that we generated for our dataset. Note that the distributions of statements in Fig. 7c, e are similar because MDS was applied using cosine dissimilarities, and the clustering for K-medoids and DBSCAN were performed using this dissimilarity measure. Consequently, Fig. 7a is the only one with a different pattern for clustering statements due to Euclidean distances in K-means. To ease conducting a visual analysis, the statements in Figs. 7a, c, e are colored according to the assigned clusters. Figure 8 illustrates the obtained clustering results arranged chronologically using the same color encoding of (Fig. 7a, c, e).

Using the values for and *MinPts* described in (“Methodology and technical background”) for DBSCAN, eight statements were considered noises, shown as black points in (Fig. 7e). More specifically, these statements are (1) September 2005, (2) March 2006, (3) May 2006, (4) August 2007, (5) September 2007, (6) October 2008, (7) December 2008, and (8) January 2009. Figure 8 shows that 7 out of 8 statements deemed as noise by DBSCAN in Fig. 7e were issued during the years in which the Great Recession elapsed.

The clusters for statements in Figs. 7a, c are well separated with a few exceptions, like the January 2009 statement in Fig. 7a and the October 2008 statement in Fig. 7c. However, the clustering results for DBSCAN in Fig. 7e differ considerably from the others. Figure 7e shows clusters that are not as clearly separated as those of Fig. 7a, c. This might be due to the consideration of density in DBSCAN instead of a fixed number of clusters. From Fig. 3, it is noticeable that the latest statements are more similar to previous ones, which agrees with the larger cluster retrieved by DBSCAN in (Fig. 7e).

Figure 7b, d, f show the silhouette plots (Rousseeuw 1987) for the clusters produced by K-means, K-medoids, and DBSCAN algorithms. Each cluster is colored in a particular color to distinguish it from other clusters. The vertical dashed red color represents the average silhouette score for the clustering. The statements deemed as noise by DBSCAN (i.e., the black points) were not included in its silhouette plot.

The average silhouette score is 0*.*423 for DBSCAN in Fig. 7f, which is lower than that for K-medoids and higher than K-means. Nonetheless, the silhouette plots in Fig. 7b, f suggest that despite the higher average silhouette score for DBSCAN, it presents an inferior clustering result. This can be seen in the more significant negative silhouette scores in Fig. 7f compared to those in Fig. 7b.

One interesting finding is that for all clustering results of K-means and K-medoids, statements within clusters are arranged chronologically for all statements, as shown in Fig. 8. This implies that even though occasionally there are significant drops in cosine similarities between consecutive statements (see Fig. 3), a statement issued at time *t* will have their most similar statements issued around *t*.

Note that all clustering techniques were applied to the original 20-dimensional space and not the bi-dimensional embedding for MDS. Hence, the fact that the colored statements in Fig. 7 follow a reasonable configuration in both the Euclidean and cosine versions of MDS shows that a clustering approach is appropriate. Furthermore, we found evidence showing that Doc2Vec is able to generate meaningful vector representations of the FOMC post-meeting statements. This is encouraging for researchers from both computer science and economics fields as other complex techniques from the NLP community could be used for more complex NLP downstream tasks.

## Conclusions and future work

In this work, we performed a similarity analysis of the Federal Reserve statements. We used two datasets: the first contains 79 statements from 2005 to 2014, and the second contains 10 statements from September 2019 to July 2020. We explored the effect of the Great Recession in the similarity of statements using the first dataset, while the second is used for exploring the effect of the COVID-19 pandemic.

Following the approach of Acosta and Meade (2015), we calculated the TF-IDF features for the first dataset. We also generated the Doc2Vec document embeddings and computed the cosine similarities between consecutive statements. Our results show that statements became more similar close to the end of this period, with significant drops in cosine similarity during the first years, which mostly coincides with the years in which the Great Recession occurred.

We conducted the same cosine similarity analysis using our second dataset, where COVID-19 is addressed first in the FOMC post-meeting statements. We found that the cosine similarities of statements decrease notoriously when the pandemic is addressed and stabilizes for statements close to July 2020. More precisely, for the Doc2Vec embeddings, the most significant drops in similarity during the pandemic are 10.8% bigger than those of the Great Recession. The fact that such drops in similarity are present during the Great Recession and the COVID-19 pandemic shows that the vector representation of statements obtained using Doc2Vec helps capture the variations in the content of the statements when a disruptive event occurs while keeping at the same time a relatively low dimensionality when compared to TF-IDF. This is especially useful for solving tasks using machine learning methods affected by the Curse of Dimensionality.

We then explored a clustering approach using K-means, K-medoids, and DBSCAN to find patterns within the first dataset. Our findings show that although there are significant drops in the cosine similarities of consecutive statements, they are grouped chronologically. Furthermore, statements deemed as noise by DBSCAN were issued primarily during the period of the Great Recession.

Researchers have applied several NLP techniques exploring their usage in economics and social sciences in general, as presented in the Related Work section. Experiments related to these methods on the FOMC statements are limited; hence, the potential of FOMC’s document embeddings in more complex NLP tasks is still unexplored. As mentioned in previous sections, the motivation of our clustering exercise was to probe the viability of such embeddings in more complicated downstream NLP tasks. Our results of the clustering experiments show that the Doc2Vec document embeddings can successfully capture the semantics of the FOMC statements; this is evident as most of the sets of clusters obtained for the three algorithms used in this work were aligned chronologically.

Moreover, the application of the DBSCAN algorithm revealed that most of the statements deemed as noisy observations were issued during, or around, the period of the Great Recession. The latter supports the idea that document embeddings can capture the semantics of disruptive events in the FOMC statements. The successful clustering exercise opens opportunities to explore the application of embeddings of the FOMC statements in cutting-edge NLP tasks beyond this paper’s scope, such as text summarization, sentiment analysis, and question answering systems, among others.

The analysis presented in our study may have its limitations. Throughout the years, the FOMC post-meeting statements have followed a very similar template regarding the way the content is presented. However, at the same time, the statements have been evolving, changing the underlying communication strategy^1^. For example, the statements have become longer as the number of sentences and words per sentence increases. Because of this, changes in the writing style could make an essential contribution to the variance of the similarities that we calculated, although such changes in the style have been demonstrated to be infrequent and gradual over time. Because of this, a limitation of our study could be the difficulty of telling whether such variance of the similarities comes from a change in the writing style or an actual disruptive event.

Another limitation is the apparent lack of the capture of the context of these two disruptive events. The Great Recession and the COVID-19 pandemic occurred in different contexts and have distinct consequences from an economic perspective. The document embeddings models applied in this study do not capture such contexts.

As future work, it will be interesting to explore how the FOMC post-meeting statements and their similarities evolve over time. Also, the inclusion of more sophisticated linguistic analysis could provide a deeper insight into how exactly the content of the statements is affected by disruptive events such as the ones considered in this work. Also, the interpretability of the results of the clustering exercise suggests that the document embeddings generated for the FOMC statements can be potentially used in more complex NLP downstream tasks. This finding allows future researchers to explore various directions in the usage of vector representations of the statements. Finally, we want to acknowledge the possibility of conducting a more fine-grained study on the FOMC statements. For instance, labelling the paragraphs according to their content to effectively track them over time. Such annotated dataset would enable us to perform the analysis presented in this study focusing on specific topics (e.g., economic outlook) instead of the comprehensive perspective of the statements that we explored in our work. Since the construction of such annotated dataset is time-consuming and out of the scope of this paper, we identify it as future work.

## Footnotes

1 https://www.stlouisfed.org/education/feducation-video-series/episode-4-understanding-an-fomc-statement . This link contains a lecture about the changing nature of FOMC postmeeting statements.
